# Safety, Feasibility, and Biomarker Effects of High-Dose Vitamin D Supplementation Among Women at High Risk for Breast Cancer

**DOI:** 10.19070/2326-3350-SI01001

**Published:** 2015-02-23

**Authors:** KD Crew, T Xiao, PS Thomas, MB Terry, M Maurer, K Kalinsky, S Feldman, L Brafman, SR Refice, DL Hershman

**Affiliations:** 1 Department of Medicine, College of Physicians and Surgeons, Columbia University, New York, NY, USA; 2 Department of Epidemiology, Mailman School of Public Health, Columbia University, New York, NY, USA; 3 Herbert Irving Comprehensive Cancer Center, Columbia University, New York, NY, USA; 4 Department of Clinical Cancer Prevention, MD Anderson Cancer Center, Houston, TX, USA; 5 Department of Surgery, College of Physicians and Surgeons, Columbia University, New York, NY, USA

**Keywords:** Vitamin D, Breast Cancer, Chemoprevention, Biomarkers

## Abstract

Vitamin D deficiency is a potentially modifiable risk factor that may be targeted for breast cancer prevention. We examined the safety, feasibility, and biomarker effects of high-dose vitamin D among women at high risk for breast cancer. Forty high-risk women, defined as a 5-year breast cancer risk ≥1.67% per the Gail model, lobular or ductal carcinoma *in situ*, were assigned to a 1-year intervention of vitamin D3 20,000 IU or 30,000 IU weekly. Participants were monitored for toxicity every 3 months, underwent serial blood draws at baseline, 6 and 12 months, and a digital mammogram at baseline and 12 months. Biomarker endpoints included serum 25-hydroxyvitamin D [25(OH)D], 1,25-dihydroxyvitamin D [1,25(OH)_2_D], parathyroid hormone (PTH), insulin-like growth factor (IGF-1), IGF binding protein (IGFBP-3), and mammographic density (MD) using Cumulus software. From November 2007 to January 2011, we enrolled 40 women; 37 were evaluable at 6 months and 30 at 12 months. One patient was taken off study for hypercalciuria; otherwise, the intervention was well tolerated. From baseline to 12 months, mean serum 25(OH)D and 1,25(OH)_2_D rose from 20.0 to 46.9 ng/ml and 69.7 to 98.1 pg/ml, respectively (p<0.01). Serum PTH decreased by 12% at 6 months and IGF-1/IGFBP-3 ratio decreased by 4.3% at 12 months (p<0.05). There was no significant change in MD regardless of menopausal status or dose level. We demonstrated that 1 year of high-dose vitamin D3 was associated with a significant increase in circulating vitamin D levels and favorable effects on IGF signaling, but no significant change in MD.

## Introduction

Vitamin D deficiency is often defined as circulating 25-hydroxyvitamin D [25(OH)D] levels of less than 20 ng/ml [1–3]. Sufficient blood concentrations of 25(OH)D generally focus on bone health, with a common definition of an optimal level of ≥32 ng/ml, which maximally suppresses serum parathyroid hormone (PTH). By these standards, the majority of the U.S. population is vitamin D insufficient or deficient.

Vitamin D has diverse biological effects relevant to carcinogenesis, including known cross-talk between the vitamin D receptor (VDR) and insulin-like growth factor (IGF) signaling pathways [4]. Based upon observational data, women with serum 25(OH) D levels greater than 40–50 ng/ml had a 50% lower risk of breast cancer compared to women with vitamin D deficiency [5].

The current recommended dietary allowance (RDA) of vitamin D is 600 IU per day for those 70 years or younger, but some argue that this dose is too low to provide a public health benefit. Oral daily intake of 1000 IU of vitamin D increases circulating 25(OH) D levels by about 10 ng/ml [6]. Given the high prevalence of vitamin D deficiency in the general population, in order to raise serum 25(OH)D above 40–50 ng/ml, the putative target level for breast cancer risk reduction, individuals would have to consume about 3000–4000 IU daily, which remains below the current upper safety limit set by the Institute of Medicine (IOM). Vitamin D toxicities, including hypercalcemia, hypercalciuria, bone demineralization, and nephrocalcinosis, are rare and generally only occur when serum 25(OH)D rises above 150 ng/ml [7].

The relationship between vitamin D status and mammographic density (MD), a strong predictor of breast cancer risk, remains unclear [8]. MD refers to the relative proportions of radiolucent fat and radiodense epithelial and stromal tissue [9,10] and may serve as a useful intermediate biomarker for breast cancer risk assessment in investigations of potential chemopreventive agents. Cross-sectional studies evaluating the association between vitamin D intake and MD observed an inverse association among premenopausal women, particularly with high serum IGF-1 and low serum IGF binding protein-3 (IGFBP-3) [11,12]. However, there is limited data on the biologic effects of vitamin D supplementation for breast cancer prevention in human intervention trials.

We conducted a pilot study in 40 women at high-risk for breast cancer development, who were assigned to a 1-year intervention of vitamin D3 (cholecalciferol) 20,000 IU or 30,000 IU weekly. We hypothesized that vitamin D supplementation modulates serum-based biomarkers of vitamin D metabolism and IGF signaling and decreases MD, a strong intermediate biomarker of breast cancer risk.

## Material and Methods

### Subjects

We conducted two pilot studies at the breast center of Columbia University Medical Center (CUMC) in New York, NY. Twenty high-risk premenopausal women were assigned to a 1-year intervention of vitamin D3 20,000 IU weekly (N=10) or 30,000 IU weekly (N=10) (NCT00976339). In a concurrent study, 20 high-risk postmenopausal women were randomized to vitamin D3 20,000 IU weekly vs. 30,000 IU weekly for 1 year (NCT00859651). We combined the results from the two trials, which were approved by the institutional review board at CUMC. All participants provided written informed consent in English or Spanish.

High risk for breast cancer was defined as having a predicted 5-year risk of invasive breast cancer according to the Breast Cancer Risk Assessment Tool (BCRAT) or Gail model (www.cancer.gov/bcrisktool) of 1.67% or greater, lobular carcinoma in situ (LCIS), or resected ductal carcinoma in situ (DCIS). Both premenopausal and postmenopausal women were included in this analysis. Postmenopausal status was defined as >6 months since last menstrual period, prior bilateral oophorectomy, or serum follicle-stimulating hormone (FSH) values consistent with institutional normal values for postmenopausal status (>20 mIU/mL).

Other eligibility criteria included:

1. age 21 years or older;
2. normal baseline clinical breast exam and mammogram;
3. baseline mammographic density (MD) greater than or equal to 25% as assessed qualitatively by the Breast Imaging-Reporting and Data System (BIRADS) classification (2=scattered fibroglandular densities, 25–50%; 3=heterogeneously dense, 51–75%; 4=extremely dense, >75%);
4. baseline serum 25(OH)D less than 32 ng/ml;
5. prior use of a selective estrogen receptor modulator (SERM), tamoxifen or raloxifene, allowed as long as the SERM was discontinued at least 28 days prior to enrollment;
6. willingness to allow collection of blood for biomarker analysis and banking;
7. at least one evaluable breast for MD assessment, defined as no bilateral mastectomies, bilateral breast implants, or prior radiation to the contralateral breast (among women with DCIS);
8. willingness to not take calcium or vitamin D supplements during the 1-year intervention; however, up to 1000 mg of calcium supplementation was allowed for postmenopausal women;
9. normal serum calcium;
10. no history of kidney stones;
11. adequate renal and hepatic function with serum creatinine, bilirubin, transaminases, and alkaline phosphatase <2.0x the institutional upper limit of normal (IULN);
12. no hypersensitivity reactions to vitamin D;
13. Zubrod performance status of 0 or 1;
14. not pregnant or nursing;
15. no significant medical or psychiatric condition that would preclude study completion.

### Study Design and Intervention

Premenopausal women participated in a non-randomized open-label trial, in which the first 10 participants were assigned to vitamin D3 (cholecalciferol) 30,000 IU (3 capsules) weekly, then the next 10 participants received vitamin D3 20,000 IU (2 capsules) weekly for 1 year. Postmenopausal women were randomized to vitamin D3 30,000 IU (3 active capsules) weekly vs. vitamin D3 20,000 IU (2 active capsules + 1 placebo capsule) weekly for 1 year. Only in the postmenopausal study were participants and investigators blinded to vitamin D dose level. The doses of vitamin D3 20,000 IU weekly (~2800 IU daily) and 30,000 IU weekly (~4300 IU daily) were chosen based upon the predicted dose levels needed to raise serum 25(OH)D to the putative target level of 40–50 ng/ml [6]. The vitamin D3 capsules (10,000 IU each) and matching placebo were supplied by PROPHARMA, LLC (BTR Group, Inc., Pittsfield, IL) under an investigational new drug (IND 77,391) application.

The primary objectives were to determine the safety and feasibility of high-dose vitamin D for 1 year among high-risk women. All participants were evaluable for toxicity from the time of their first dose of study drug. Safety was assessed by monitoring routine clinical and laboratory parameters, including serum calcium, albumin, and creatinine and spot urine calcium and creatinine, every 3 months for the duration of the study.

### Correlative Studies

Secondary objectives were to investigate the biologic effects of vitamin D supplementation on blood-based and image-based biomarkers. Blood samples were collected at baseline, 6 and 12 months for measurement of serum 25(OH)D, 1,25-dihydroxyvitamin D [1,25(OH)_2_D], PTH, IGF-1, and IGFBP-3. Serum 25(OH)D and 1, 25(OH)_2_D were assessed by an HPLC method that selectively measures vitamin D2 and D3, as previously described [13]. Serum PTH was measured by a standard 2-site immunoradiometric assay (Scantibodies Laboratory, Santee, CA) that detects only whole PTH,1-84 and does not measure inactive PTH fragment [14]. Serum IGF-1 and IGFBP-3 levels were assayed by ELISA analysis with reagents from Diagnostic Systems Laboratories. The interassay coefficient of variation for serum 25(OH)D and 1, 25(OH)_2_D was <10%. Inter-and intra-assay precision for PTH were 6.3% and 2.8%, respectively. For IGF-1 and IGFBP-3, intra-assay precision were 3.5% and 1.0%, respectively. All blood samples were analyzed in batches by blinded personnel.

Bilateral digital mammograms were conducted at baseline and after the 12-month intervention. Due to changes in MD with the menstrual cycle, mammograms were obtained within 10 days of the start of menstrual bleeding in premenopausal women. Mammographic percent density (proportion of the breast with dense tissue) from cranio-caudal views was assessed using semi-automated methods by the Cumulus software [15]. All MD readings were conducted by an investigator (MBT) blinded to treatment assignment and the timing of the mammograms (baseline or 12 months); we randomized digitized images from the same women within the same batch.

### Statistical Analysis

The percentage of participants with each toxicity was compared between the treatment groups using a Fisher’s exact test of proportions at a two-sided 0.05 level of significance. Descriptive statistics were performed for each of the biomarker endpoints for the two dose levels and for both dose levels combined. We calculated the means (standard deviation, SD), medians (range), and percent change from baseline for each biomarker. Paired t-tests and 2-sample t-tests (using raw data or log-transformed data from the perspective of normality assumption) were used to compare within-group and between-group differences for the two vitamin D dose levels, respectively. For the secondary biomarker endpoints, we had 80% power to detect a difference of 0.51SD after vitamin D supplementation (both dose levels combined), using a paired t-test at the 0.05 significance level and assuming a 20% unevaluable rate. Repeated measure analysis of variance (ANOVA) was used with a time interaction term using dose level as the main effect. We then conducted subgroup analyses stratifying by vitamin D dose level, baseline serum 25(OH)D (<20 ng/ml vs. 20–32 ng/ml), and menopausal status. In addition, correlation coefficients and multivariable linear regression with adjustment for known confounders, such as age, race, body mass index (BMI), menopausal status, and baseline MD, were used to determine the relationship between MD and blood biomarkers. All statistical analyses were conducted using SAS 9.2 (SAS Institute, Cary, NC) and p<0.05 was considered statistically significant in all analyses.

## Results

**Figure 1:**
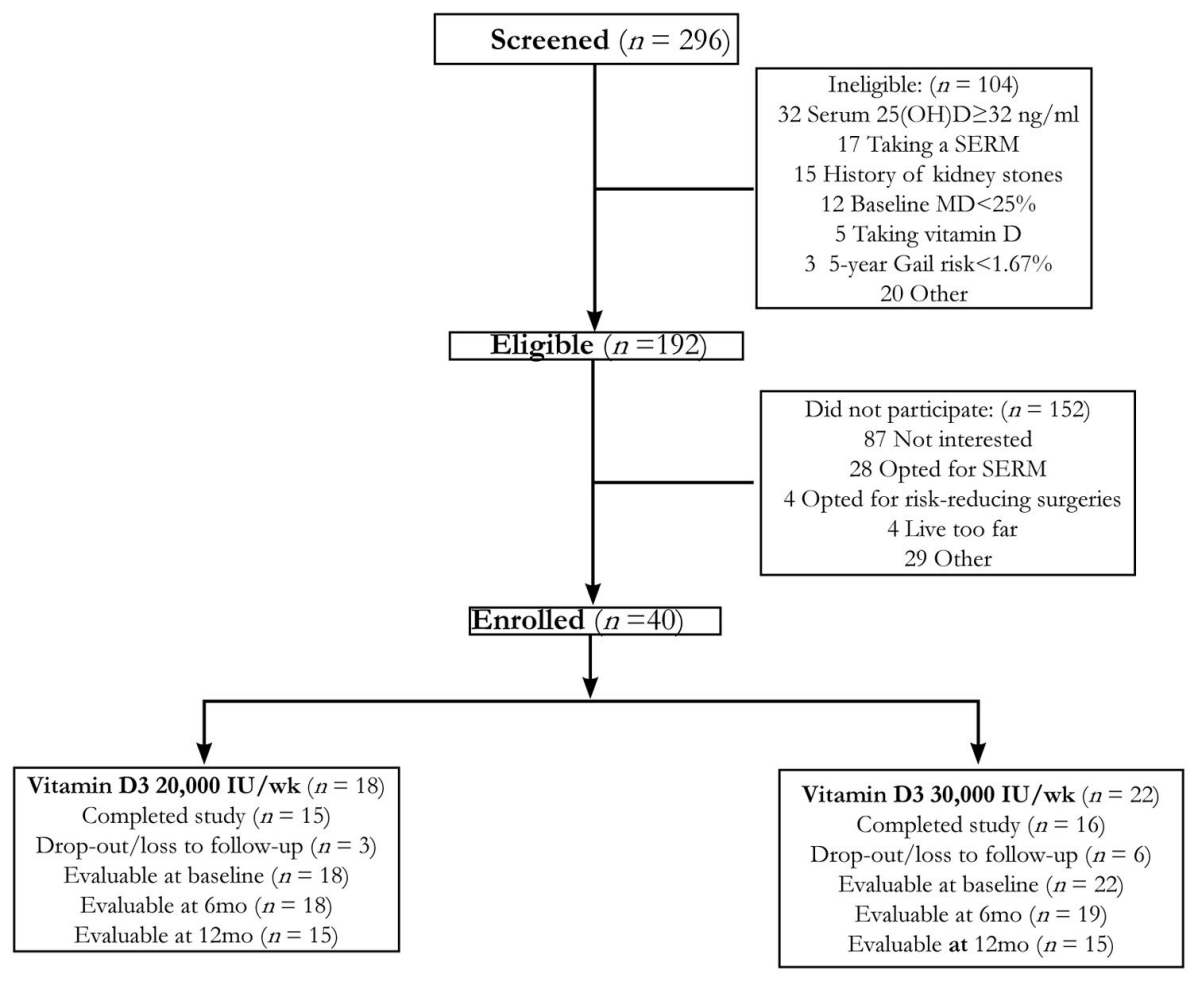
Flow diagram for subjects who were accrued into the study. Abbreviations: 25(OH)D, 25-hydroxyvitamin D; MD, mammographic density; SERM, selective estrogen receptor modulator.

From November 2007 to January 2011, we screened 296 women for participation and 192 (65%) were eligible (Figure 1). The main reasons for ineligibility included serum 25(OH)D ≥ 32 ng/ml (31%), currently taking a SERM (16%) or vitamin D supplement (5%), history of kidney stones (14%), and baseline MD <25% (12%). Of the 192 eligible women, 40 (21%) enrolled including 20 premenopausal and 20 postmenopausal women. Eighteen women were assigned to a 1-year intervention of vitamin D3 20,000 IU weekly and 22 women received a dose of 30,000 IU weekly. There was a higher rate of drop-out/loss to follow-up at the higher dose level (27%) compared to the lower dose of vitamin D (17%). Of the 40 women enrolled, 37 were evaluable at 6 months and 30 at 12 months.

Baseline characteristics of the enrolled participants are summarized in Table 1. The median age was 50 years (range, 37–73 years), median BMI was 26.6 kg/m^2^ (range, 20.0–39.6 kg/m^2^), and median serum 25(OH)D was 19.9 ng/ml (range, 9.4–30.4 ng/ml). The study population was predominately Non-Hispanic white and Hispanic, 45% and 50%, respectively. Half the women met high-risk criteria according to the Gail model and the remainder was diagnosed with either LCIS or DCIS. Sixty-five percent of women had a baseline MD that was mildly dense (BIRADS 2=scattered fibroglandular densities, 25–50%). At baseline, premenopausal women compared to postmenopausal women tended to have a lower median BMI (25.2 vs. 28.3 kg/m^2^), higher mammographic percent density (27% vs. 15%), and lower serum 25(OH)D (15.9 vs. 22.6 ng/ml).

No grade 3 or higher adverse events occurred at either dose level of vitamin D (Table 2). More fatigue, gastrointestinal and dermatologic toxicities were observed with vitamin D3 30,000 IU weekly compared to 20,000 IU weekly, but only constipation was statistically significant (6 vs. 0, p=0.024). There was one episode of grade 1 hypercalciuria (defined as spot urine calcium/creatinine ratio >0.37) at the 30,000 IU dose level, which required stopping study drug. However, there were no episodes of hypercalcemia or hypervitaminosis D at either dose level. Based upon self-reported vitamin and supplement use at baseline, 7 out of 20 postmenopausal women reported taking calcium supplements. However, during the 1-year vitamin D intervention, serum calcium levels did not change from baseline (mean, 9.3 mg/dl; range, 8.7–10.0 mg/dl) to 6 months (mean, 9.4 mg/dl; range, 8.5–10.1 mg/dl) or 12 months (mean, 9.4 mg/dl; range, 8.5–10.5 mg/dl).

**Figure 2:**
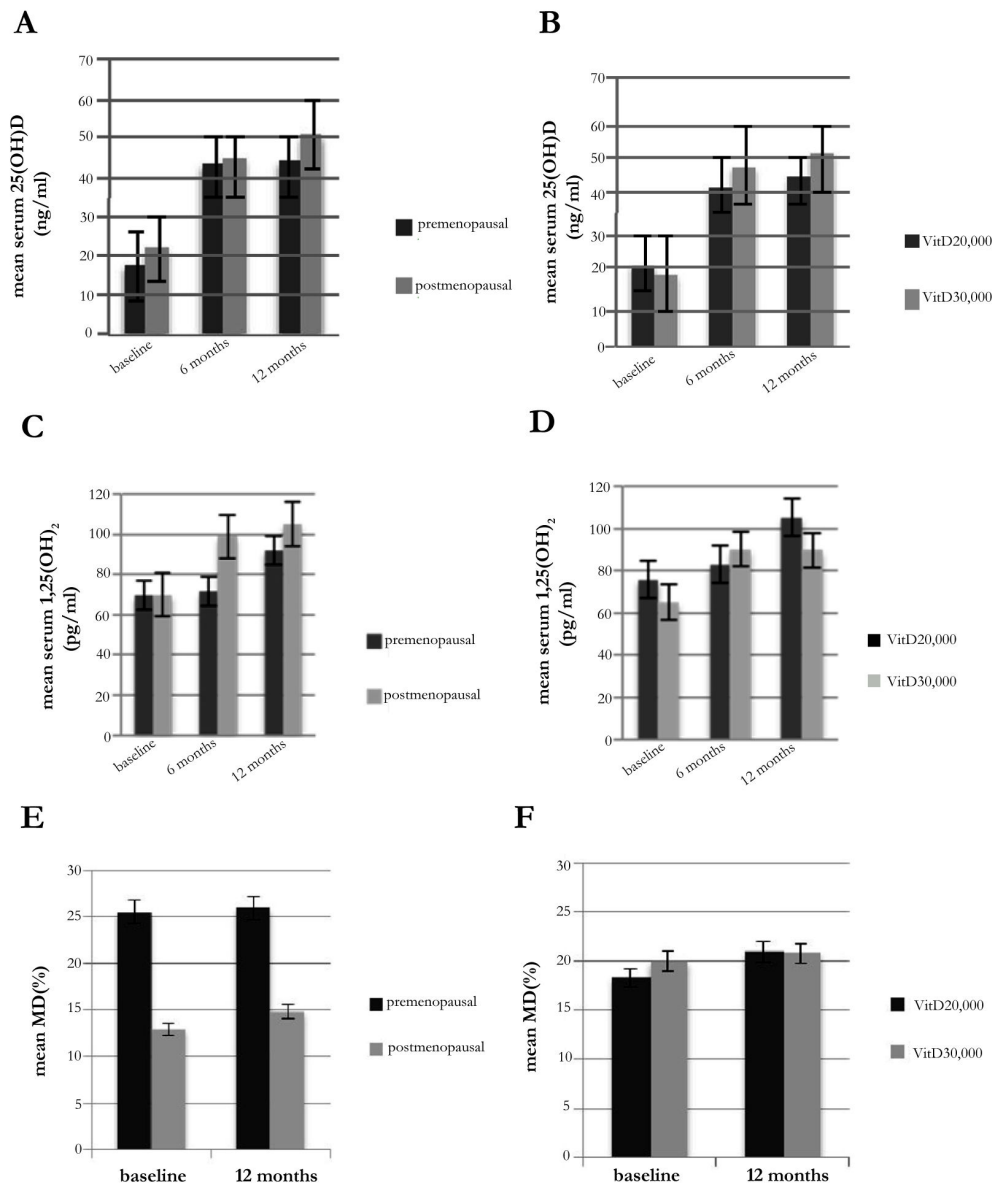
(A, B) Mean serum 25-hydroxyvitamin D [25(OH)D], (C, D) serum 1,25-dihydroxyvitamin D [1,25(OH)_2_D], and (E, F) mammographic density (MD) stratified by menopausal status and vitamin D dose level.

Combining the results of both dose levels at baseline, 6 and 12 months (Table 3), mean serum 25(OH)D rose from 20.0 to 43.8 to 46.8 ng/ml, respectively (p<0.001); and mean serum 1,25(OH)_2_D increased from 69.7 to 86.8 to 98.1 pg/ml, respectively (p<0.001). These changes did not differ by vitamin D dose level (Figure 2). After 12 months, 26 (87%) of the 30 evaluable women reached the target ≥ 40 level of serum 25(OH)D ng/ml. Compared to baseline, serum PTH decreased by 12% at 6 months (p=0.004), but this difference was no longer significant at 12 months. We observed a 5% decrease in serum IGF-1 at 6 and 12 months, which did not reach statistical significance. However, serum IGF-1/IGFBP-3 ratio significantly decreased by 4.3% at 12 months (p=0.011). When we conducted stratified analysis by baseline serum 25(OH)D level (<20 ng/ml vs. 20–32 ng/ml), we noted a similar increase in serum 25(OH)D at 12 months (mean absolute change of 28.6 ng/ml vs. 24.3 ng/ml, respectively). However, the decrease in IGF-1/IGFBP-3 ratio was restricted to those with a baseline serum 25(OH)D <20 ng/ml (mean absolute change at 12 months of −3.3 × 10^−3^, p=0.003).

After 1 year of high-dose vitamin D, there was no significant change in mean percent density from baseline to 12 months (19.2% vs. 20.8%, p=0.537), regardless of menopausal status, vitamin D dose level, or baseline serum 25(OH)D (Figure 2). At 12 months, MD was inversely correlated with serum 25(OH)D (correlation coefficient=−0.45, p=0.02) and positively associated with serum IGF-1 (correlation coefficient=0.54, p=0.005). However, these associations were not significant after adjustment for age, race, BMI, and menopausal status (data not shown). In multivariable analysis, change in MD did not correlate with changes in any blood biomarkers after adjusting for age, race, BMI, menopausal status, and baseline MD (data not shown).

## Discussion

Overall, high-dose vitamin D was well-tolerated with no grade 3 or higher toxicities and only 1 episode of hypercalciuria at the 30,000 IU weekly dose level. At both dose levels of vitamin D, there was a significant increase in serum 25(OH)D at 6 and 12 months with over 85% achieving the putative target level of ≥40 ng/ml. We observed a favorable effect on circulating IGF-1/IGFBP-3 ratio, but no significant change in MD with 1 year of vitamin D3 (cholecalciferol).

Cholecalciferol is a vitamin D precursor, which gets hydroxylated in the liver to 25(OH)D (calcidiol), the main circulating form, and then hydroxylated in the kidney by 1α-hydroxylase to 1,25(OH)_2_D (calcitriol), the most active metabolite [16]. Many extrarenal tissues, including the breast, also express 1α-hydroxylase to locally activate vitamin D, which has paracrine and autocrine effects in these tissues [17]. Circulating 25(OH)D is the substrate for conversion to 1,25(OH)_2_D in target tissues and may be the limiting factor in local activation of vitamin D [18].

In women given standard doses of vitamin D 400–600 IU daily, no substantial change in serum 25(OH)D was observed and the majority remained in the insufficient range [19,20]. Recent clinical trials demonstrated increases in vitamin D levels only with high-dose supplementation. We observed a significant increase in serum 25(OH)D and 1,25(OH)_2_D at both dose levels of vitamin D. The mean increase in serum 25(OH)D of 20–30 ng/ml was in line with what was anticipated with daily oral intake of 2000–3000 IU daily of vitamin D3 [6].

Numerous observational studies have reported an inverse association between vitamin D status, including circulating 25(OH)D levels, and breast cancer risk. In a meta-analysis of observational studies, an increase in serum 25(OH)D by 20 ng/ml was inversely associated with breast cancer risk, with a summary relative risk (RR)=0.73 (95% confidence interval (CI)=0.60–0.88) [21]. However, nearly all of these studies reported a single measurement of 25(OH)D. There have been no prospective studies evaluating the effect of change in vitamin D level with serial measurements over time on breast cancer risk.

On the other hand, the Institute of Medicine (IOM) has raised concerns about negative health effects of circulating 25(OH)D above 50 ng/ml [22]. Some observational studies have found U-shaped relationships between cancer incidence rates and serum 25(OH)D [23–25], although the statistical power to investigate risk at very high levels of 25(OH)D in these studies was very low. Vitamin D toxicities, including hypercalcemia and hyperciuria, are rare and tend to occur when serum 25(OH)D rises above 150 ng/ml. A 17% increased incidence of kidney stones was observed with calcium and vitamin D compared to placebo in the Women’s Health Initiative (WHI) randomized controlled trial, 2.5% vs. 2.1%, respectively [26]. A review of clinical trial data on the safety of high-dose vitamin D suggested that daily doses as high as 10,000 IU are safe [27]. We observed slightly more fatigue, rash, and gastrointestinal toxicities with vitamin D3 30,000 IU weekly compared to 20,000 IU weekly, but no significant differences in the biomarker endpoints.

Preclinical studies support various antitumor effects of vitamin D in breast cancer. The vitamin D receptor (VDR) is a ligand-dependent transcription factor that regulates a wide range of cellular processes central to cancer development, including apoptosis, cell proliferation, differentiation, angiogenesis, and metastasis [28]. Specific pathways affected include IGF signaling, causing inhibition of the MAPK and ERK pathways [4]. IGFs are peptide hormones with anti-apoptotic and mitogenic effects [29–31] and IGFBPs, of which IGFBP-3 is the most abundant form, block IGF-1 from binding to its receptor [32]. Several epidemiologic studies reported a significant positive correlation between circulating IGF-1 and breast cancer risk, particularly among premenopausal women [33–35]. Although 1 year of high-dose vitamin D supplementation did not significantly alter serum IGF-1 and IGFBP-3, there was a favorable effect on IGF-1/IGFBP-3 ratio at 12 months. The change in IGF-1/IGFBP-3 ratio was statistically significant but modest (mean absolute change, 1.5 × 10^−3^; percentage change, 4.3%). Whether this change is clinically relevant remains to be seen, however, comparable differences were seen between breast cancer cases and age-matched unaffected controls [36]. Since we did not adjust for multiple comparisons for the secondary biomarker endpoints, these results need to be interpreted with caution and confirmed in larger studies.

In terms of mammographic density (MD), women in the highest quartile of MD demonstrated a 4-to-6-fold increase in breast cancer risk compared to women in the lowest quartile [10]. In addition to qualitative assessment with BIRADS classification, MD can now be assessed on a continuous scale using computer-assisted techniques [15]. Anti-estrogens, such as tamoxifen, have been shown to significantly reduce MD within 12–18 months of initiation [37]; however, the effects of non-hormonal interventions on MD remain uncertain.

A recent systematic review of 14 observational studies examining the association between vitamin D and MD yielded inconsistent results [8]. Much of the association was limited to premenopausal women [11], particularly among those with high circulating IGF-1 and low IGFBP-3 levels [12], suggesting that vitamin D may modulate MD and breast cancer risk via IGF signaling. We previously reported an inverse association between serum 25(OH) D and mammographic dense area in the late summer and fall months, when peak levels of circulating vitamin D occur [38].

In this intervention trial, we did not see a significant change in MD after 1 year of high-dose vitamin D3, although we only had sufficient statistical power to examine an absolute change of 7–8%. MD correlated with serum 25(OH)D and IGF-1 at 12 months; however, these associations were no longer significant after adjustment for known confounders. Possible explanations for these null findings include the small sample size, the need for longer follow-up, and the relatively low baseline MD in our study population. In a prior study, greater declines in percent density were observed among women with higher baseline MD [39]. In addition, the potential cancer protective effect of vitamin D may not be mediated via changes in MD. Larger studies with longer follow-up will be needed to evaluate the effects of vitamin D supplementation on MD and other intermediate markers of breast cancer risk.

An analysis of randomized controlled trials of vitamin D (300 to 2000 IU daily) with or without calcium supplementation and primary fracture outcomes demonstrated that all-cause mortality was reduced by 7% (hazard ratio [HR]=0.93, 95% CI=0.87–0.99), but without sufficient evidence to support vitamin D for cancer prevention [40, 41]. Of note, most of the trial participants were postmenopausal women over the age of 65. In the WHI, over 36,000 postmenopausal women were randomized to calcium plus vitamin D 400 IU daily or placebo, with no difference in breast cancer incidence after a mean follow-up of 7 years [20]. Among WHI participants, no significant difference in MD after a year of vitamin D supplementation was reported [42]. However, the ratio of mean breast density comparing calcium and vitamin D to placebo was 0.67 (95% CI=0.41–1.07) with ≥80% study drug compliance and no hormone replacement therapy use. In a randomized controlled trial of calcium and vitamin D 1100 IU daily for 4 years in 1,179 postmenopausal women, a 60% reduction in overall cancer incidence was found compared to placebo [43]. Neither of these trials distinguished between the effects of calcium and vitamin D. The effects of higher doses of vitamin D supplementation on breast cancer incidence, particularly among premenopausal or high-risk women, are still unknown. More recent trials assessing moderate to high doses of vitamin D to prevent cancer and other chronic diseases are currently ongoing [44].

We demonstrated the safety and feasibility of enrolling high-risk women to an intervention trial of high-dose vitamin D. However, we needed to screen nearly 300 women to enroll 40 participants over a 3-year period at our institution. The most common reason for ineligibility was baseline serum 25(OH)D ≥32 ng/ml or current vitamin D supplement use.

Increasing awareness of vitamin D deficiency and adoption of high-dose supplementation may diminish the feasibility of conducting vitamin D intervention trials. For example, Cescon et al. found that over 80% of patients with newly diagnosed breast cancer reported current vitamin D supplement use and over 70% already had adequate levels of serum 25(OH)D [45].

To our knowledge, this is the first study to report the effects of high-dose vitamin D supplementation on biomarkers of breast cancer risk among high-risk women. Strengths of this study included the relatively long 1-year intervention, testing of two different dose levels of vitamin D, collection of serial blood samples and mammograms for biomarker analyses, and the racially/ethnically diverse study population. Over half the participants in our trial were from racial/ethnic minority groups, which have a higher prevalence of vitamin D deficiency compared to non-Hispanic whites [46]. The main weaknesses were the relatively small sample size and higher than anticipated drop-out rate (25%).

The biomarker effects of high-dose vitamin D among high-risk premenopausal women are currently under investigation. Two ongoing trials are evaluating the effects of vitamin D 2000 IU daily or 20,000 IU weekly for 1 year on change in MD among premenopausal women with increased MD or at high risk for breast cancer development (NCT01224678 and NCT01097278). These studies include serial blood draws and breast tissue sampling, which will add to our understanding of the biologic effects of vitamin D on serum and breast tissue-based biomarkers.

Vitamin D deficiency has become a shared concern among physicians, who often routinely screen for vitamin D deficiency and recommend supplementation. In the 2010 IOM report addressing vitamin D supplementation [22], the RDA for vitamin D was raised from 400 to 600 IU daily for persons 70 years and younger and the upper safety limit for healthy individuals from 2000 to 4000 IU daily. However, based upon the current literature, the IOM concluded that there was insufficient evidence to recommend vitamin D supplementation for cancer prevention (www.IOM.edu/vitaminD). Despite promising preclinical and observational data, we must await the results of rigorously conducted randomized controlled trials before making broad recommendations of vitamin D supplementation for breast cancer prevention.

## Figures and Tables

**Table 1:** Participant characteristics at baseline

Characteristics	Vitamin D3 20,000 IU/week *(N=18)*	Vitamin D3 30,000 IU/week *(N=22)*	Total (N=40)

Median age, years (range)	50.0 (45–73)	50.5 (37–69)	50 (37–73)

Menopausal status, *N* (%)			
Premenopausal	10 (56)	10 (45)	20 (50)
Postmenopausal	8 (44)	12 (55)	20 (50)

Race/Ethnicity, *N* (%)			
Non-Hispanic White	9 (50)	9 (41)	18 (45)
Hispanic	7 (39)	13 (59)	20 (50)
Non-Hispanic Black	1 (5.5)	-	1 (2.5)
Asian	1 (5.5)	-	1 (2.5)

Median body mass index, kg/m^2^ (range)	24.6 (20.0–38.8)	28.9 (20.2–39.6)	26.6 (20.0–39.6)

High-risk category, *N* (%)			
5-year breast cancer risk ≥1.67% *	8 (45)	12 (55)	20 (50)
Lobular carcinoma in situ	6 (33)	4 (18)	10 (25)
Ductal carcinoma in situ	4 (22)	6 (27)	10 (25)

Mammographic density by BIRADS score, N (%)			
2 (scattered fibroglandular densities)	13 (72)	13 (59)	26 (65)
3 (heterogeneously dense)	4 (22)	9 (41)	13 (33)
4 (extremely dense)	1 (6)	0	1 (2)

Serum 25(OH)D, N (%)			
<20 ng/ml	7 (39)	13 (59)	20 (50)
20–32 ng/ml	11 (61)	9 (41)	20 (50)

Abbreviation: 25(OH)D, 25-hydroxyvitamin D; BIRADS, Breast Imaging-Reporting and Data System.

* According to the Breast Cancer Risk Assessment Tool (BCRAT) or Gail model.

**Table 2:** Adverse events by vitamin D dose level. Only toxicities that were at least possibly related to study drug are listed.

Any Toxicity	Vitamin D 20,000 IU/week (N=18)			Vitamin D 30,000 IU/week (N=22)			P-value*

	Grade 1	Grade 2	Total N (%)	Grade 1	Grade 2	Total N (%)	

*Cardiovascular*							
Hypertension	0	1	1 (6)	0	0	0	0.450

*Constitutional*							
Fatigue	1	0	1 (6)	6	0	6 (27)	0.105
Weight gain	1	0	1 (6)	0	0	0	0.450

Dermatologic							
Dry skin	0	0	0	1	0	1 (5)	1.000
Rash	1	0	1 (6)	4	1	5 (23)	0.197

*Gastrointestinal*							
Abdominal distension	0	0	0	2	0	2 (9)	0.492
Abdominal pain	2	0	2 (11)	2	1	3 (14)	1.000
Constipation	0	0	0	5	1	6 (27)	0.024
Diarrhea	0	0	0	1	1	2 (9)	0.492
Nausea	0	0	0	4	0	4 (18)	0.114

*Metabolic*							
Hypercalciuria	0	0	0	1	0	1 (5)	1.000

*Musculoskeletal*							
Musculoskeletal pain	1	5	6 (33)	5	2	7 (32)	1.000

*Neurologic*							
Headache	1	0	1 (6)	1	0	1 (5)	1.000

*Psychiatric*							
Insomnia	1	0	1 (6)	0	0	0	0.450

* Comparison using Fisher’s exact test.

**Table 3:** Biomarker effects of a 1-year intervention of vitamin D3 20,000 IU weekly (N=18) and 30,000 IU weekly (N=22) among high-risk women.

Biomarker	Baseline (N=40)	6 months (N=37)	12 months (N=30)

***Serum 25(OH)D, ng/ml***			

Mean (SD)	20.0 (6.0)	43.8 (8.2)	46.8 (9.8)
Median (range)	19.9 (9.4–30.4)	42.7 (26.9–62.1)	46.8 (25.7–67.2)
Percentage change		+119%	+134%
P-value, paired t-test		P<0.001	P<0.001
P-value, ANOVA			P<0.001

***Serum 1,25(OH)_2_D, pg/ml***			

Mean (SD)	69.7 (21.6)	86.8 (24.1)	98.1 (34.0)
Median (range)	69.8 (33.2–116.3)	88.1 (46.8–141.9)	92.6 (13.7–156.7)
Percentage change		+24.50%	40.70%
P-value^a^		P=0.003	P<0.001
P-value^b^			P<0.001

***Serum PTH, pg/ml***			

Mean (SD)	35.0 (11.0)	29.2 (9.4)	31.9 (14.7)
Median (range)	33.2 (17.7–67.9)	28.8 (10.2–49.2)	28.0 (13.0–80.4)
Percentage change		−12.0%	−8.8%
P-value^a,^*		P=0.004	P=0.272
P-value^b^			P= 0.098

***Serum IGF-1, ng/ml***			

Mean (SD)	127.9 (39.6)	121.1 (40.1)	121.6 (28.9)
Median (range)	121.3 (52.5–257.7)	121.0 (47.1–246.3)	127.1 (41.0–171.1)
Percentage change		−5.3%	−4.9%
P-value^a,^*		P=0.239	P=0.201
P-value^b^			P= 0.685

***Serum IGFBP-3, αg/ml***			

Mean (SD)	3.75 (0.73)	3.58 (0.67)	3.72 (0.68)
Median (range)	3.76 (2.43–5.17)	3.49 (2.36–5.24)	3.70 (2.21–4.86)
Percentage change		−4.5%	+0.8%
P-value^a^		P=0.277	P=0.816
P-value^b^			P= 0.539

***Serum IGF-1/IGFBP-3 (x10^−3^)***			

Mean (SD)	34.2 (7.8)	33.8 (8.5)	32.7 (6.8)
Median (range)	35.1 (18.7–51.0)	31.5 (19.4–57.1)	34.2 (18.6–46.4)
Percentage change		−1.1%	−4.30%
P-value^a^		P=0.668	P=0.011
P-value^b^			P= 0.774

***Percent density, %***			

Mean (SD)	19.2 (15.4)		20.8 (14.6)
Median (range)	14.5 (0.9–60.4)		19.9 (2.0–60.6)
Percentage change			8.30%
P-value^a,^*			P=0.537
